# A Systematic Review of Typhoid Fever Occurrence in Africa

**DOI:** 10.1093/cid/ciz525

**Published:** 2019-10-30

**Authors:** Jong-Hoon Kim, Justin Im, Prerana Parajulee, Marianne Holm, Ligia Maria Cruz Espinoza, Nimesh Poudyal, Ondari D Mogeni, Florian Marks

**Affiliations:** 1 Public Health, Access, and Vaccine Epidemiology (PAVE) Unit, International Vaccine Institute, Seoul, Korea; 2 Department of Medicine, University of Cambridge, United Kingdom

**Keywords:** typhoid fever, *S. Typhi*, Africa, blood culture, burden of disease

## Abstract

**Background:**

Our current understanding of the burden and distribution of typhoid fever in Africa relies on extrapolation of data from a small number of population-based incidence rate estimates. However, many other records on the occurrence of typhoid fever are available, and those records contain information that may enrich our understanding of the epidemiology of the disease as well as secular trends in reporting by country and over time.

**Methods:**

We conducted a systematic review of typhoid fever occurrence in Africa, published in PubMed, Embase, and ProMED (Program for Monitoring Emerging Diseases).

**Results:**

At least one episode of culture-confirmed typhoid fever was reported in 42 of 57 African countries during 1900–2018. The number of reports on typhoid fever has increased over time in Africa and was highly heterogeneous between countries and over time. Outbreaks of typhoid fever were reported in 15 countries, with their frequency and size increasing over time.

**Conclusions:**

Efforts should be made to leverage existing typhoid data, for example, by incorporating them into models for estimating the burden and distribution of typhoid fever.

Typhoid fever is an invasive bacterial infection caused by *Salmonella enterica* serovar Typhi. It is believed that >10 million clinical *Salmonella* Typhi infections arise each year in low- and lower-middle-income countries, of which three million occur in Africa [1–3]. Although the majority of typhoid cases arise in Asia [4], recent observations in Africa imply that the burden of disease is also substantial [1, 2]. Surveillance conducted at 13 sites in 10 countries in sub-Saharan Africa between 2010 and 2014 showed that the incidence rate of typhoid fever was as high as 383 (95% confidence interval, 274–535) per 100 000 person-years in one country [2].

Our contemporary knowledge concerning the distribution and incidence of typhoid fever in Africa relies on extrapolation of data from several small-sized population-based studies reporting incidence rate estimates [1–3]. While prospective, population-based studies remain the most reliable source of data on typhoid fever incidence, such studies are highly resource intensive. The majority of countries in Africa lack data on typhoid incidence estimates from prospective studies and will need to make critical decisions about the introduction of typhoid conjugate vaccines (TCVs) in the absence of these data.

Although population-based typhoid incidence studies from Africa remain sparse, many other forms of data on the occurrence of typhoid fever are available, and such data may enrich our understanding of the epidemiology of the disease [5, 6]. To address gaps in our knowledge of the incidence of typhoid fever in Africa, we conducted a systematic review of typhoid fever reports in the scientific literature and ProMED (Program for Monitoring Emerging Diseases).

## METHOD

### Search and Data Sources

Data sources for typhoid fever occurrence included peer-reviewed research articles (from PubMed and Embase) and reports from ProMED, an internet-based reporting system where infectious disease occurrence is identified in media reports, official reports, online summaries, and other similar platforms. We conducted a systematic literature search using iterations of the term typhoid fever, including “typhoid,” “*S*. Typhi,” “*Salmonella* Typhi,” or “enteric fever” mentioned in the full text. We limited articles relevant to the African continent by requiring the mention of at least one African country in the text. For example, to retrieve references related to typhoid fever in Algeria, we used the following search term: (“typhoid” OR “*S.* Typhi” OR “*Salmonella* Typhi” OR “enteric fever”) AND “Algeria.”

### Eligibility Criteria and Study Selection

We included studies where time, location, and diagnostic method for the occurrence of human typhoid cases are clearly described. All types of articles including reports of sporadic cases, outbreak investigation, cross-sectional surveys, clinical trials, and longitudinal surveillance conducted in Africa were considered eligible. We included reports of culture-confirmed typhoid fever where *Salmonella* Typhi was isolated from blood, stool, or bone marrow as the primary evidence but also reports of typhoid fever confirmed through serologic tests (e.g., Widal test or enzyme-linked immunosorbent assay [ELISA]) or polymerase chain reaction (PCR) or suspected clinically (e.g., ileal perforation) for comparison.

We did not limit the search based on date of publication and included articles written in English and French. In addition to full-text articles, we included articles for which only abstracts were available as long as time, location, and diagnostic method for the occurrence of human typhoid cases were clearly described. We excluded the studies that do not report original occurrence of typhoid fever in humans. For instance, many retrieved articles concern molecular biological characteristics of preexisting isolates (eg, susceptibility to antimicrobials or other medicinal plants) and were therefore excluded. Some studies were excluded because they report the occurrence of a *Salmonella* infection without providing information about their serotype. Other studies were excluded because they report a survey or an analysis of the existing data, but do not report novel occurrence of typhoid cases. Where there was discordance among the two reviewers, the first author made a determination after discussions.

### Data Extraction, Study Variables, and Analytic Approach

Two authors (J.-H. K. and P. P.) reviewed the literature and extracted the data. In particular, the following variables were extracted: year and country of the typhoid occurrence, year of reporting, diagnostic method, and the number of typhoid cases reported. In reports of typhoid cases from observations that span multiple years without further details broken down by year, we assumed that at least 1 episode of typhoid fever case occurred each year.

We defined a typhoid fever occurrence primarily as a report of at least 1 episode of culture-confirmed typhoid fever where *Salmonella* Typhi was isolated at a given space and time excluding any duplicate reports from the same cohort. Reports of typhoid fever confirmed through serologic tests (e.g., Widal test or ELISA) or PCR, or suspected clinically (e.g., ileal perforation), were also included for secondary analysis. For imported cases, we assumed that the typhoid episode occurred in the country in which the infection was presumed to have been acquired (according to the report). If the imported cases led to local transmission, we assumed that typhoid occurred in both countries. Outbreaks were defined according to the author of the respective study.

### Risk of Bias

Difference in language use across Africa may introduce a bias in our study if we limit the source of information to one specific language. We therefore included studies written in French as well as those written in English because French is the predominant language in many West and Central African countries. Use of diagnostic methods with low specificity is another factor that may introduce a bias in our study. Typhoid fever confirmed by clinical diagnosis or Widal tests will likely include false-positive cases. This may lead to an overestimation, and its extent may vary by time and location. To avoid this potential bias, we included typhoid fever confirmed by culturing blood, stool, urine, or bone marrow samples in the primary analysis and included the rest in the secondary analysis for comparison. On the other hand, isolation of *Salmonella* Typhi from blood, a definitive diagnosis of typhoid fever, is known to have <60% sensitivity [7], leading to underestimation.

### Synthesis of Result

The primary outcome of interest is the geotemporal occurrence of typhoid fever in the African continent. Occurrences of typhoid fever from different reports were summed to calculate the number of reports by country by year. Occurrences by the same cohort (eg, reported from the same hospital) in the same year were counted only once whereas occurrences from the different cohorts were regarded as separate occurrence events.

## RESULTS

### Overview of Evidence of Typhoid Fever Occurrence

**Figure 1:**
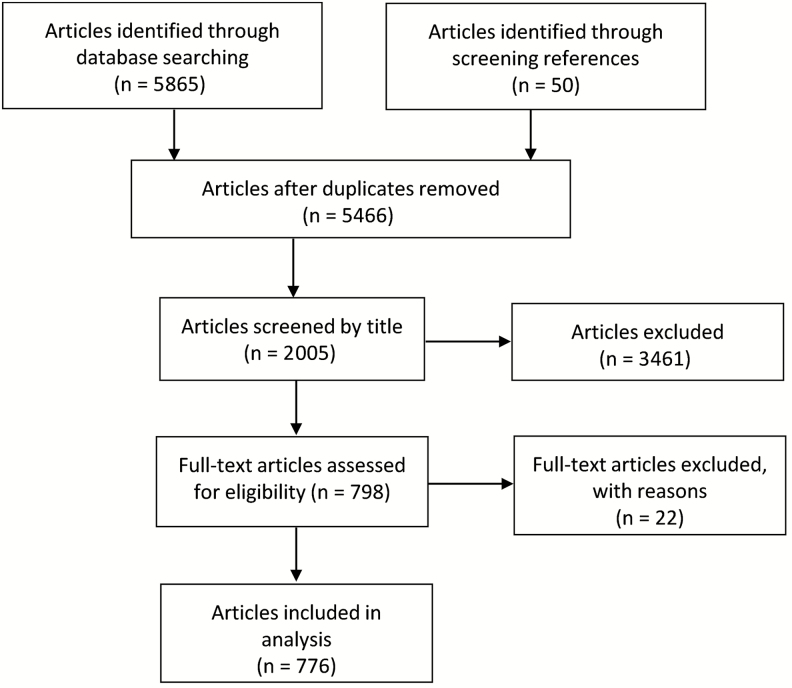
. Flowchart of the literature review procedure.

**Figure 2.**
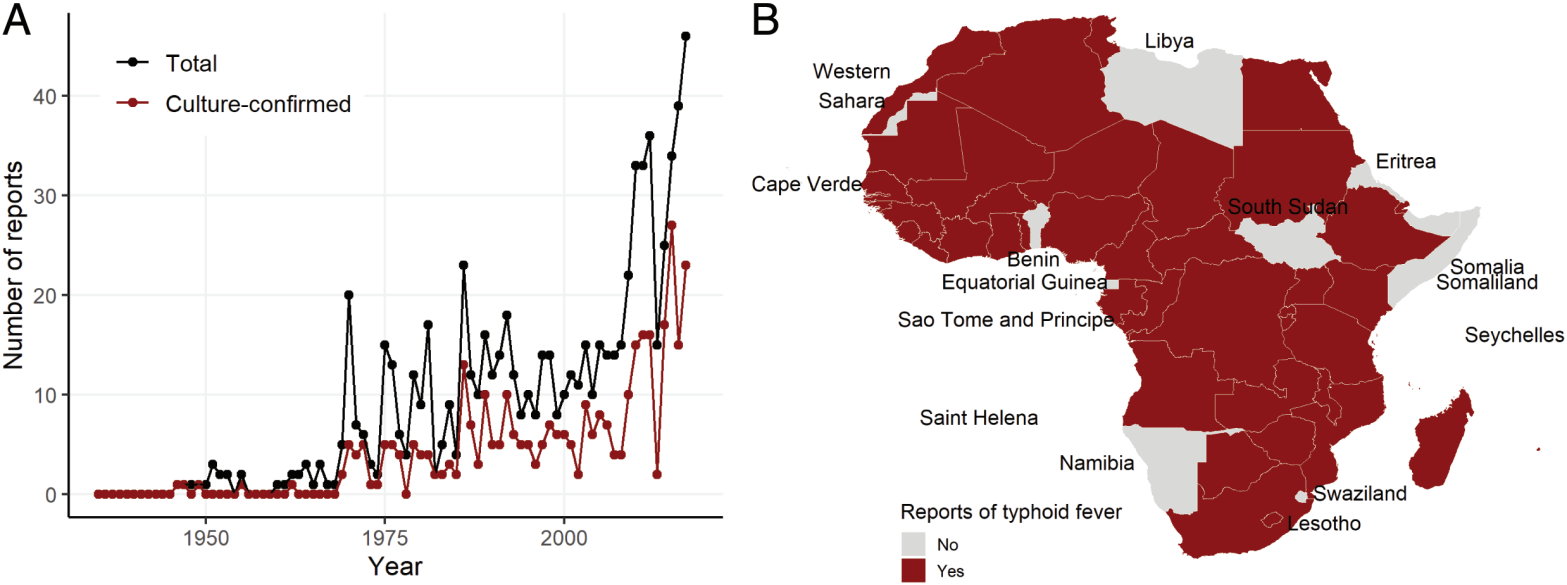
*A*, Number of typhoid fever occurrence reports from Africa over time. Total reports are inclusive of articles reporting typhoid fever occurrence as confirmed by Widal test, clinical definition, or culture confirmation. *B*, Map of countries with at least one report of typhoid fever.

We screened 1537, 3914, and 15 articles from PubMed, Embase, and ProMED, respectively, and selected 609, 161, and 6 unique references, respectively, for further analysis (Figure 1). Overall, 42 of 57 African countries have reported at least 1 typhoid fever occurrence, and the total number of reports tended to increase over time (Figure 2). Fifteen countries for which we did not find reports on typhoid fever occurrence include those with low population density (Libya and Namibia), small population size (São Tomé and Principe, Seychelles, and Cape Verde), and high political instability (Libya, Somalia, and South Sudan). Diagnoses of typhoid fever were reported in studies based on the isolation of *Salmonella* Typhi cultured from blood, stool, urine, rectal swab, or cerebrospinal fluid sample (n = 335), Widal test (n = 44), or clinical diagnosis of a typhoid fever–related complication such as intestinal perforation (n = 398) (Supplementary Table 1). In a few instances, molecular diagnostics, such as PCR (n = 6) and TaqMan Gene Expression Array Cards (Applied Biosystems, Foster City, California) (n = 1), or antibody-capture ELISA (n = 1) were used to diagnose typhoid fever. For this analysis, these occurrences were considered to be equivalent to blood culture confirmation. The largest number of articles reporting typhoid fever occurrence originated in Nigeria (n = 158), followed by Egypt (n = 95), South Africa (n = 72), and Ghana (n = 67). Only 1 report was available for each of Botswana, Chad, Djibouti, Guinea, and Mauritania.

### Spatial and Temporal Distribution of Typhoid Fever Occurrence

**Figure 3.**
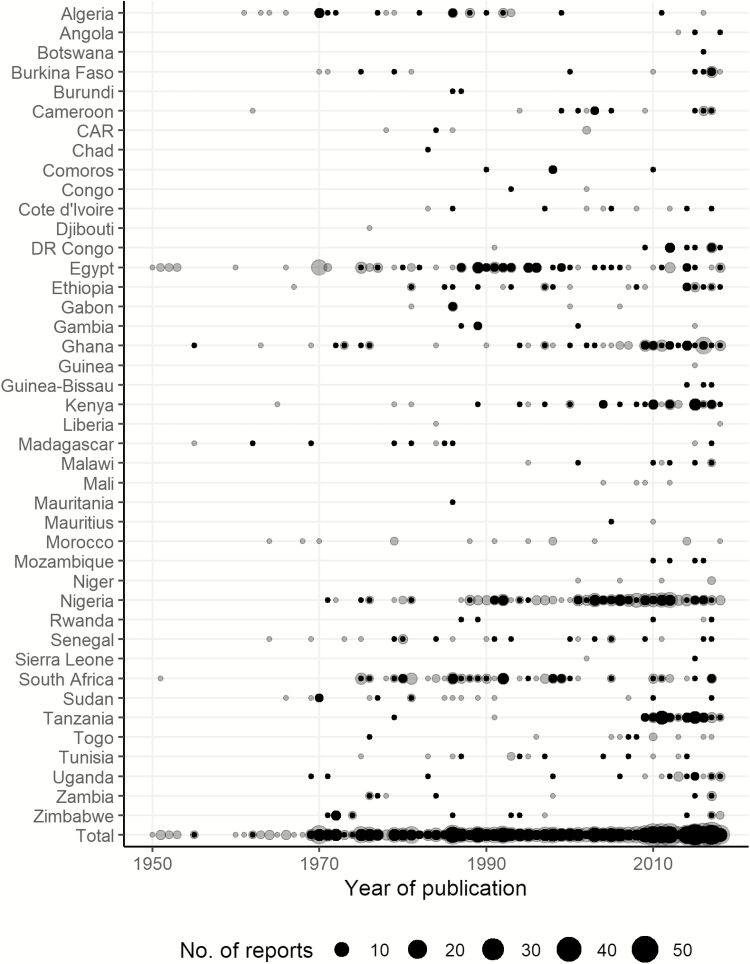
Number of reports on typhoid fever by country and year. Studies that were published before 1950 (n = 5) were omitted for better display. Gray dots indicate the number of typhoid fever reports (including Widal-confirmed, clinical, and culture-confirmed); black dots indicate reports of culture-confirmed typhoid fever. Abbreviations: CAR, Central African Republic; DR Congo, Democratic Republic of the Congo.

The number of reports was highly heterogeneous by year and country (Figure 3). Typhoid fever had not been reported until 2010 in Angola, Guinea, Guinea-Bissau, and Mozambique and not until 1990 in Mali and Malawi, whereas in Egypt, the first occurrence of typhoid fever was published as early as 1901 [8, 9]. No country has reported typhoid continuously since 1950, and most countries had periods during which typhoid fever has not been reported. Nigeria reported typhoid cases for most years since 1980, whereas the largest gap between published reports of typhoid was between 1986 and 2011 (25 years) in Madagascar. Typhoid occurrences were reported in Burundi, Chad, Djibouti, Liberia, and Mauritania before 1990; however, until the time of this publication, no additional reports of typhoid fever were found. In terms of African subregions, the majority of the typhoid occurrences in 2000 and afterward arose in Western Africa followed by Eastern Africa (Supplementary Figure 1).

### Outbreaks of Typhoid Fever

**Figure 4.**
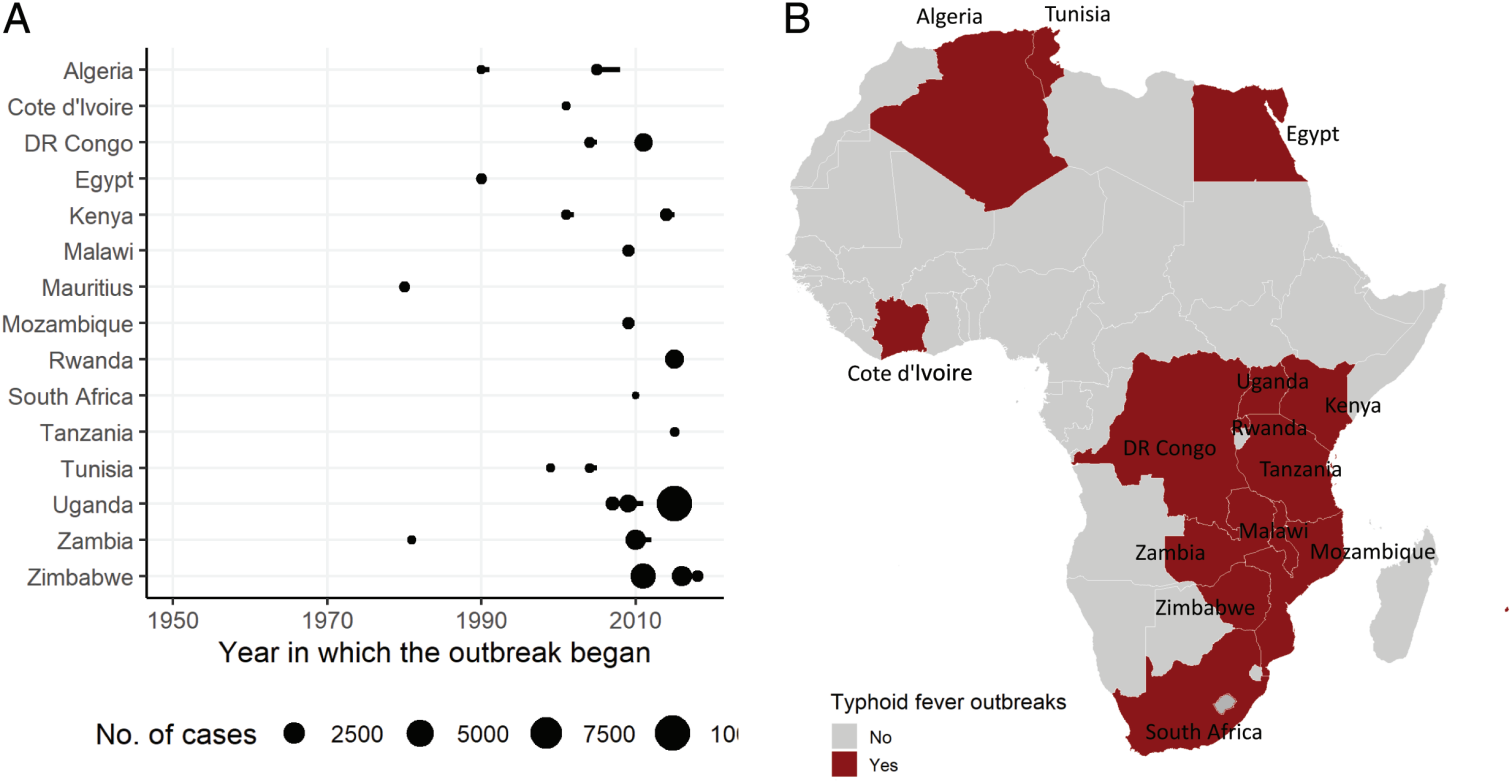
Reported outbreaks of typhoid fever in Africa since 1950. *A*, The horizontal bars attached to some of the dots indicate the duration (year) of the outbreak. *B*, The map highlights spatial distribution of countries where outbreaks of typhoid fever have been reported since 2000. Abbreviation: DR Congo, Democratic Republic of the Congo.

Outbreaks of typhoid fever were reported in 15 countries since 1950 (in 17 countries since 1900 (Table 1), and the majority have occurred in the southeastern part of the African continent (Figure 4). The frequency of reported outbreaks of typhoid fever and the number of people affected appear to have increased over time. The earliest reports were outbreaks during the Anglo-Boer War in South Africa between 1899 and 1902 [8, 9], and the most recent record was in January 2018 when a sudden increase in typhoid fever cases (n > 200) was observed in Harare, Zimbabwe [10]. The largest outbreak was in Kampala, Uganda, between February and June 2015, where a total of 10 230 suspected cases were associated with a typhoid-confirmed breakout [11], although the magnitude of the outbreak in South Africa in 1900 might have been larger [8]. Recent outbreaks have occurred mostly in East Africa: Moyale, Kenya (December 2014–January 2015) [11]; Kampala, Uganda (February–June 2015) [12]; Kigoma, Tanzania (May 2015) [13]; and Kirehe, Rwanda (October 2015–January 2016) [14].

**Table 1.** Outbreaks of Typhoid Fever in Africa

Country	District or Subpopulation	Time	No. of Cases	Source
Algeria	Fourchi	Jun–Aug 2005	90 cases (blood culture-confirmed)	[24]
	Fourchi	Oct 2006	60 cases (blood culture-confirmed)	[24]
	Ain Kercha	Aug–Oct 2007	14 cases (blood culture-confirmed)	[24]
	Ain Kercha	Aug–Dec 2008	14 cases (blood culture-confirmed)	[24]
	Oran^a^	1978	NA	[25]
	Dergana (suburb of Algiers)	Nov 1990–Apr 1991	34 (blood culture-or serology-confirmed in 30 cases)	[26]
Côte d’Ivoire	French troops in Abidjan	Aug–Sep 2001	24 cases (14 confirmed cases)	[27]
DRC	Kikwit, Bandundu	2011	1430 cases (blood- [6/16] and stool [7/13] culture-positive)	[28]
	Bwamanda, Sud-Ubangi, Equateur	Nov 2011–May 2012	18 blood culture confirmed	[29]
	Kinshasa	Oct 2004–Jan 2005	144 peritonitis patients (11 blood culture–positive cases of 16 tested)	[30]
Egypt	Gharbeya Governorate	Nov–Dec 1990	133 cases (blood-, stool-, and urine culture-positive in 26%, 9%, and 6% of cases, respectively)	[31]
Gambia	Manduar	8 weeks, 1989^b^	26 cases (7/9 blood culture-positive and 22/24 Widal tests were positive)	[32]
Kenya	Mandera	Nov 1943–Jan 1944	13 cases (*S.* Typhi isolated from 1 case)	[33]
	Thika, Embu, and Nairobi	Jan 2001–Dec 2002	102 (3, 14, and 85 respectively) *S*. Typhi isolates	[34]
	Moyale in Marsabit County	Dec 2014–Jan 2015	317 cases (Widal test-positive in 155 cases; stool culture-positive in 71/188 cases tested)	[11]
Malawi / Mozambique	Neno (Malawi) and Tsangano (Mozambique)	Mar–Nov 2009	303 cases (214 suspected [clinical symptoms], 43 probable [rapid test positive], 46 confirmed [blood culture-positive or fever with stool culture-positive])	[35]
Malawi	Neno and Mwanza	2009–Dec 2012	850 cases and 43 deaths (*S*. Typhi isolated)	[36]
	Dowa	Jan–Apr 2013	146 cases and 1 death (*S*. Typhi isolated)	[36]
	Mchinji	Oct 2014	NA	[36]
	Kasungu	Jan 2015	NA	[36]
	Neno	Jul 2016	139 cases (10th week, 3 blood culture-confirmed)	[37]
Mauritius	NA	1980	126 cases	[38]
Rwanda	Mahama sector, Kirehe district	Oct 2015–Jan 2016	1663 cases (*S*. Typhi isolated)	[14]
South Africa	Dannhauser, Newcastle, and Nqutu districts, KwaZulu-Natal	Apr–Jun 1991	6 *S*. Typhi isolates	[39]
	Bloemfontein, Orange Free State	1990	4959 cases (British troops)	[8]
	Cape Town	Dec 1900	NA	[9]
	Grassy Park, Cape Town	Apr–May 1978	69 cases (*S*. Typhi isolated in 61 cases; blood [n = 24], stool [n = 53], or urine [n = 8])	[40]
	Guguletu, Cape Town	1978	10 cases (*S*. Typhi isolated in 9 cases)	[40]
	Delmas	2005^b^	2900 cases (>400 confirmed cases)	[41]
	Delmas	1993	1000 cases	[41]
	Pretoria	Apr–May 2010	8 *S*. Typhi isolates	[42]
	Delmas	1996^b^, possibly 1993 [41]	55 cases (46 seropositive cases)	[43]
	Stykraal, Tswaing, Mooiplaas, Vlakplaas	Nov 2017	>60 cases (*S*. Typhi from the water near the affected villages)	[44]
Sudan	Mellit and 38 villages in its vicinity	1964	>800 cases (*S*. Typhi isolated from stool)	[45]
Tanzania	Kigoma	May 2015	60 cases (blood culture-confirmed [n = 10], TaqMan Array Card-confirmed [n = 8], and confirmed in both tests [n = 2])	[13]
Tunisia	Gabes	Nov 2004	39 cases (blood- or stool culture-confirmed)	[46]
	Gabes	Oct–Nov 2005	37 cases (blood- or stool culture-confirmed)	[46]
Uganda	Kasese district	Dec 2007–July 2009	577 cases (*S.* Typhi isolated from 27/81 cases)	[47]
	Kasese, Bundibugyo, Kabarole, other districts, and also from DRC	Aug 2009–Jan 2012	1341 cases (blood- or stool culture-confirmed in 24/154 cases tested)	[48]
	Kampala	Feb–Jun 2015	10 230 cases (TUBEX test-positive in 1038/3464 cases and blood culture-positive in 56/364 cases)	[12]
Zambia	Mansa, Luapula Province	Jan–Feb 1981	43 (blood culture-positive in 2/43 cases tested)	[49]
	Lusaka	Jan 2010–Sep 2012	2040 cases (94 *S*. Typhi isolates)	[50]
	Kalingalinga, Lusaka Province	May 2017^b^	NA	[51]
	Mazabuka and Monze, Southern Province	Nov 2017^b^	57 suspected cases (9 confirmed)	[52]
Zimbabwe	Harare	Oct 2011–Apr 2012	4233 cases (52 confirmed cases)	[53]
	Harare	Oct 2016–Mar 2017	860 cases (80 confirmed by blood or stool culture)	[54]
	Harare	Oct 2016–Dec 2016	>2200 cases (126 confirmed)	[55]
	Mbare	Oct 2017	NA	[56]
	Harare	2018	200	[10]

Abbreviations: DRC, Democratic Republic of the Congo; NA, not available; *S.* Typhi, *Salmonella enterica* serovar Typhi.

^a^This article is written in Czech and was included by the title without consulting abstract or full text.

^b^Year of publication; actual dates not available.

## DISCUSSION

Based on review of published reports, we found that typhoid fever occurrences in Africa were highly heterogeneous between countries and over time. Although the overall volume of reports of typhoid fever occurrences have increased, this augmentation in reporting does not necessarily reflect higher typhoid fever incidence rates by country or overall for the given period, and could reflect better surveillance and reporting systems Modeled estimates suggest a slight decrease in incidence since 1990 [15], whereas the frequency of reported typhoid occurrences has increased quadratically in the same period.

Existing estimates of overall incidence and spatial distributions of typhoid fever rely on (part of) population-based surveillance conducted in 20 sites in 12 countries of Africa [1–3, 16–20]. We found that more data exist concerning the transmission of typhoid fever in Africa. For example, culture-confirmed typhoid cases and outbreaks have been reported in 42 and 15 countries, respectively. We should put efforts to increasingly use these data to better understand the epidemiology of the disease and improve design disease control strategies including TCV introduction.

This analysis does not attempt to measure the true geotemporal incidence of typhoid fever in Africa, which requires systematic and comparable surveillance data reporting *per capita* incidence of disease in defined populations; such data can be subsequently extrapolated to settings without surveillance using geostatistical modeling approaches [1–3]. As reports of occurrence of typhoid fever are contingent on many factors, of which some are unrelated to the epidemiological nature of disease, the interpretation of these data should be approached with care. Consistent reporting of typhoid fever could reflect sustained endemicity of the disease, or it could simply be confounded by increases in reporting capacity despite opposing epidemiological trends (Supplementary Figure 2). Similarly, intermittent reporting could reflect periodic breakouts related to ecological risk factors or could be an artifact of temporary research studies that resulted in augmented surveillance for the duration of the study, and a rise in the number of reports could reflect improvements in health care infrastructure in general. This implies that although we could not find reports on the occurrence of typhoid fever for 15 countries, it is possible that those countries are not all typhoid-free. This absence of reporting could reflect a broader blind spot due to social and political factors [21, 22] or competing public health priorities [23] that make it challenging to detect or report the disease in the respective countries. In addition, inferences on spatial and temporal distribution of typhoid fever based on our literature review are susceptible to biases. Although our study includes studies published in both English and French, we cannot rule out the possibility that, due to the limited numbers of French journals, as compared to English ones, opportunities to publish from Francophone countries may be lower; in addition, the culture of publishing case reports in certain areas is not as strong as others. Therefore, some of the typhoid occurrences were not reported and the level of this underreporting may vary by country and year. Also, although we limited the primary evidence of typhoid fever to the microbiological isolation of *Salmonella* Typhi, the sensitivity of culturing methods is low and dependent on various factors (e.g., sample volume, prior treatment with antibiotics) [7], which introduces additional uncertainty.

## CONCLUSIONS

We have demonstrated that data on typhoid fever, not limited to blood culture–based, population-based incidence estimates, can be useful for interpreting broader geotemporal trends in typhoid epidemiology. Efforts should be made to leverage existing typhoid data, for example, by devising methods to incorporate them into models for estimating the incidence and distribution of typhoid fever. This can help us better understand the epidemiology of the disease, which will in turn help us make better-informed policy decisions such as the introduction of TCV. Although population-based surveillance provides the most reliable source of information on disease incidence, numerous surveillance studies are still necessary to patch the gap in comparable population-based typhoid fever data from Africa. Other existing forms of data we collated in this study can be used to improve the current estimates of disease incidence.

## Supplementary Data

Supplementary materials are available at *Clinical Infectious Diseases* online. Consisting of data provided by the authors to benefit the reader, the posted materials are not copyedited and are the sole responsibility of the authors, so questions or comments should be addressed to the corresponding author.

ciz525_suppl_Supplemental_data

## Notes

***Author contributions.*** J.-H. K., J. I., and F. M. conceived the study. J.-H. K. and P. P. reviewed the literature. J.-H. K., J. I., and M. H. analyzed the data. All authors discussed the results and contributed to the final manuscript.

***Financial support.*** This research was funded by The Bill & Melinda Gates Foundation [OPP1127988]. The International Vaccine Institute acknowledges its donors including the Republic of Korea and the Swedish International Development Cooperation Agency. This publication was made possible through a grant from the Bill & Melinda Gates Foundation [OPP1201031].

***Supplement sponsorship.*** This article was published as part of the supplement “Severe Typhoid Fever in Africa (SETA) Program” sponsored by the International Vaccine Institute.

***Potential conflicts of interest.*** The authors: No reported conflicts of interest. All authors have submitted the ICMJE Form for Disclosure of Potential Conflicts of Interest. Conflicts that the editors consider relevant to the content of the manuscript have been disclosed.
