# Supplementary material for: A Systematic Review of Typhoid Fever Occurrence in Africa
Source: Clin Infect Dis. 2019 Oct 30;69(Suppl 6):S492–8. doi: 10.1093/cid/ciz525 (PMC6821235; doi:10.1093/cid/ciz525)
Supplement: ciz525_suppl_Supplemental_data [file ciz525_suppl_supplemental_data.docx]

# Supplementary Material

Figure S 1. The number of reports on typhoid fever by African subregion and year. Studies that were published before 1950 (n=5) were omitted for better display. Grey dots indicate the number of typhoid reports (including Widal-confirmed, clinical, as well as culture-confirmed) while black dots indicate reports of culture-confirmed typhoid fever.


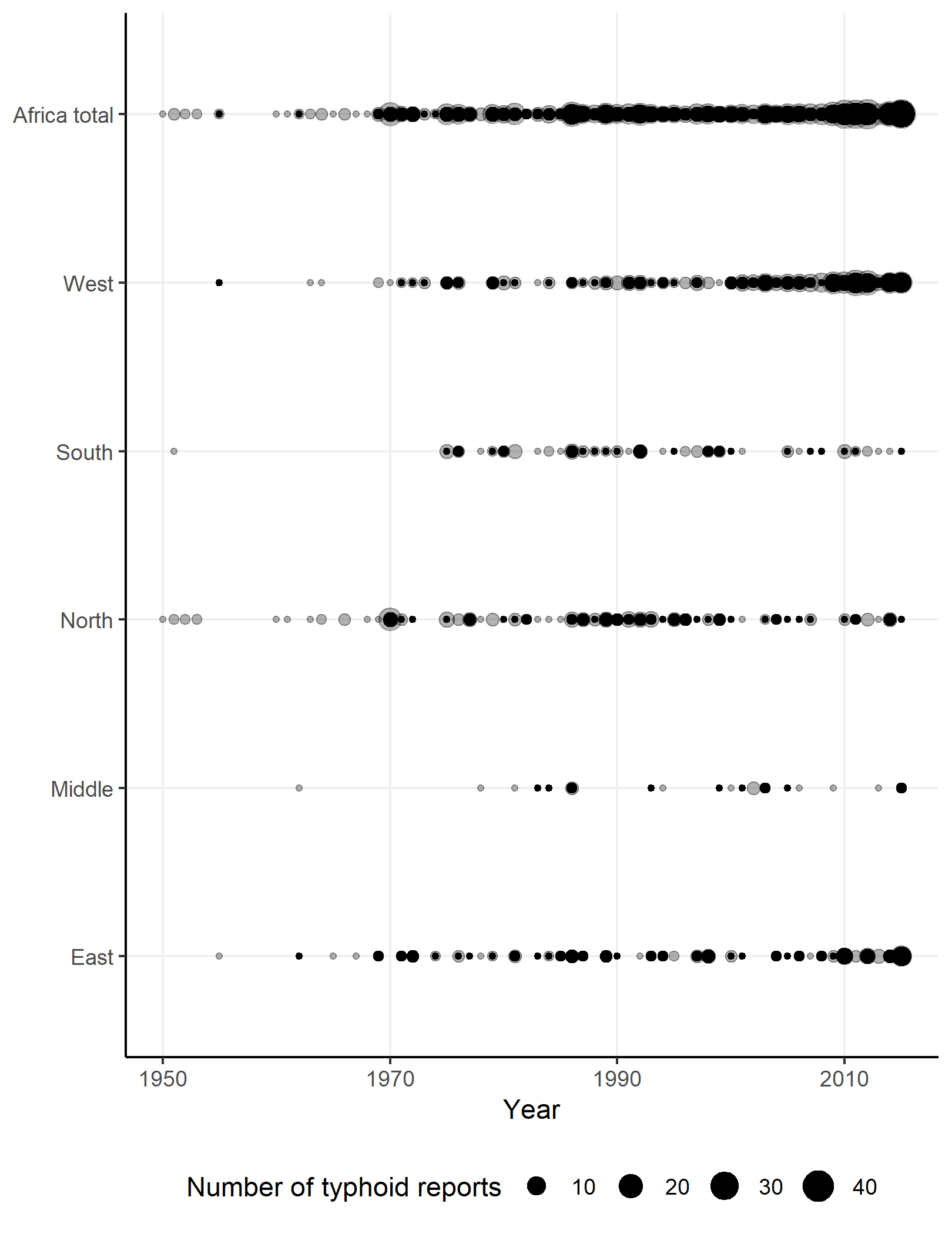


Table S1. Reports of typhoid fever occurrence in Africa

| Country | Total | Culture-confirmed (CSF) | Widal-confirmed | Clinically suspected | Total PubMed articles |
| --- | --- | --- | --- | --- | --- |
| Algeria | 28 | 15 | - | 13 | 2,844 |
| Angola | 3 | 2 | 1 | - | 927 |
| Botswana | 1 | 1 | - | - | 1,616 |
| Burkina Faso | 18 | 9 | 1 | 8 | 2,989 |
| Burundi | 2 | 2 | - | - | 621 |
| Cameroon | 15 | 6 | 1 | 8 | 5,040 |
| CAR | 5 | 1 | - | 4 | 744 |
| Chad | 1 | 1 | - | - | 685 |
| Comoros | 4 | 4 | - | - | 283 |
| Congo | 2 | 1 | - | 1 | 1,730 |
| Cote d'Ivoire | 11 | 5 | - | 6 | 2,967 |
| Djibouti | 1 | - | - | 1 | 212 |
| DR Congo | 12 | 9 | - | 3 | 3,862 |
| Egypt | 95 | 37 | 4 | 54 | 13,754 |
| Ethiopia | 23 | 13 | 3 | 7 | 10,944 |
| Gabon | 6 | 3 | - | 3 | 1,397 |
| Gambia | 5 | 4 | - | 1 | 3,005 |
| Ghana | 67 | 24 (2) | 2 | 41 | 7,223 |
| Guinea | 1 | - | - | 1 | 960 |
| Guinea-Bissau | 5 | 5 | - | - | 883 |
| Kenya | 36 | 22 (2) | 6 | 8 | 14,684 |
| Liberia | 2 | - | - | 2 | 1,110 |
| Madagascar | 9 | 6 | - | 3 | 3,212 |
| Malawi | 8 | 5 | 1 | 2 | 4,739 |
| Mali | 4 | - | 1 | 3 | 2,220 |
| Mauritania | 1 | 1 | - | - | 415 |
| Mauritius | 2 | 1 | - | 1 | 532 |
| Morocco | 14 | - | - | 14 | 5,324 |
| Mozambique | 4 | 4 | - | - | 2,155 |
| Niger | 5 | - | 2 | 3 | 1,112 |
| Nigeria | 158 | 43 (3) | 14 | 101 | 26,717 |
| Rwanda | 5 | 4 | - | 1 | 2,173 |
| Senegal | 24 | 14 | 1 | 9 | 5,463 |
| Sierra Leone | 2 | 1 | - | 1 | 1,381 |
| South Africa | 72 | 22 (1) | - | 50 | 39,131 |
| Sudan | 16 | 6 | 1 | 9 | 4,506 |
| Tanzania | 40 | 30 (1) | 3 | 7 | 10,392 |
| Togo | 11 | 3 | 1 | 7 | 1,076 |
| Tunisia | 14 | 6 | 1 | 7 | 7,690 |
| Uganda | 20 | 10 (2) | 1 | 9 | 10,965 |
| Zambia | 10 | 5 | - | 5 | 4,165 |
| Zimbabwe | 15 | 10 (1) | - | 5 | 5,513 |
| Total | 777 | 335 (12) | 44 | 398 |  |

CAR=Central African Republic; CSF=cerebrospinal fluid
